# Segmentum: a tool for copy number analysis of cancer genomes

**DOI:** 10.1186/s12859-017-1626-8

**Published:** 2017-04-13

**Authors:** Ebrahim Afyounian, Matti Annala, Matti Nykter

**Affiliations:** Faculty of Medicine and Life Sciences and BioMediTech institute, University of Tampere, Tampere, Finland

**Keywords:** Somatic copy number analysis, Loss of heterozygosity, Segmentation, Whole-genome sequencing, Cancer

## Abstract

**Background:**

Somatic alterations, including loss of heterozygosity, can affect the expression of oncogenes and tumor suppressor genes. Whole genome sequencing enables detailed characterization of such aberrations. However, due to the limitations of current high throughput sequencing technologies, this task remains challenging. Hence, accurate and reliable detection of such events is crucial for the identification of cancer-related alterations.

**Results:**

We introduce a new tool called Segmentum for determining somatic copy numbers using whole genome sequencing from paired tumor/normal samples. In our approach, read depth and B-allele fraction signals are smoothed, and double sliding windows are used to detect breakpoints, which makes our approach fast and straightforward. Because the breakpoint detection is performed simultaneously at different scales, it allows accurate detection as suggested by the evaluation results from simulated and real data. We applied Segmentum to paired tumor/normal whole genome sequencing samples from 38 patients with low-grade glioma from the TCGA dataset and were able to confirm the recurrence of copy-neutral loss of heterozygosity in chromosome 17p in low-grade astrocytoma characterized by *IDH1/2* mutation and lack of 1p/19q co-deletion, which was previously reported using SNP array data.

**Conclusions:**

Segmentum is an accurate, user-friendly tool for somatic copy number analysis of tumor samples. We demonstrate that this tool is suitable for the analysis of large cohorts, such as the TCGA dataset.

**Electronic supplementary material:**

The online version of this article (doi:10.1186/s12859-017-1626-8) contains supplementary material, which is available to authorized users.

## Background

Somatic copy number alterations (SCNA) are a group of genomic aberrations commonly observed in many cancers [1]. Copy number is the number of copies per cell of a particular gene or DNA sequence. Somatically acquired chromosomal rearrangements such as deletions and duplications may change the copy number of a gene. Consequently, the expression level of a gene is often correlated with its copy number [2] - a phenomenon known as the gene dosage effect. Loss of heterozygosity (LOH) is an event in which one of the two alleles at a heterozygous locus is lost due to segmental aneuploidy, gene conversion, mitotic recombination, or mitotic nondisjunction [3]. LOH events involving tumor suppressor genes such as *PTEN*, *RB1,* and *TP53* have been observed in many cancer. LOH may alter gene expression. For example, monoallelic expression (MAE), which is the expression of a gene from only one of two alleles in a diploid organism, is associated with LOH [3]. By analyzing a cohort of 23 triple-negative breast cancer patients, Ha et al. [3] have shown that LOH is a prominent aberration in this type of cancer, and modulates a significant portion of the transcriptome in the form of MAE. Copy-neutral LOH (cnLOH) is a specific type of LOH that occurs when the lost allele is replaced with a duplicated copy of the surviving allele, resulting in the copy number remaining unchanged. Suzuki et al. have shown recurring cnLOH at chromosome 17p (harboring *TP53* gene) in low-grade astrocytoma [4]. The altered expression of genes with allelic imbalance due to LOH events may bring about selective advantages for tumorigenesis and tumor progression. Additionally, regions with cnLOH may harbor genes with driver mutations [5]. Hence, accurate and reliable detection and characterization of events, such as SCNAs and LOH, are crucial for the identification of prospective cancer-related genes, such as tumor suppressor genes and oncogenes, and eventually for informing new approaches to treat cancer [6].

High throughput sequencing (HTS)-based SCNA detection approaches (including both whole exome sequencing (WES) and whole genome sequencing (WGS)) have become popular due to their potential for accurate copy number estimation and breakpoint detection with single nucleotide accuracy. However, the short read length of current HTS technologies makes it difficult to map some reads to unique locations in the genome. Furthermore, due to GC-content bias, GC-content-rich regions in the genome will have increased number of reads. These ambiguities make accurate estimation of coverage and consequently copy number a challenge [7]. Additionally, tumor ploidy and normal cell contamination introduce further challenges in SCNA detection [8].

HTS-based copy number analysis is, in most cases, based on read depth (RD) estimations at each genomic location and further segmentation and quantification of the RD profiles into segments of consistent copy number (Additional file 1: Table S1 for a list of SCNA tools) [9, 10]. However, such tools are only capable of detecting deletions and duplications. Recently, RD-based analysis has been augmented to identify cnLOH events by incorporating information from an alternate allele’s fraction at heterozygous single nucleotide polymorphism (SNP) positions (or B-allele fraction (BAF)). The BAF of a heterozygous SNP has an expected value of 0.5 in normal diploid cells. Deviation from 0.5 in the heterozygous SNP BAF points to an aberration. In the case of cnLOH, BAF values are expected to be either 0 or 1 in a pure tumor population. Tools such as Control-FREEC [11], Patchwork [12], and CLImAT [13] incorporate BAF data to extend SCNA detection. Control-FREEC determines the breakpoints using a least absolute shrinkage estimator (LASSO) regression. Sample ploidy is provided by the user to Control-FREEC. It also evaluates and corrects for normal cell contamination, GC-content, and mapability biases while inferring the copy number profile of a tumor genome. Patchwork performs GC and positional normalization and segments the genome using a circular binary segmentation (CBS) algorithm. It also estimates normal cell contamination and tumor ploidy. CLImAT implements corrections for GC-content and mapability bias and models the RD and BAF data with a hidden Markov model (HMM) to infer the somatic copy number variation, normal cell contamination and tumor ploidy (Additional file 1: Overview of Tools section for more details on these tools). While the above tools are well-suited for SCNA detection, their use has some limitations. Control-FREEC and Patchwork utilize computationally costly models, which leads to long analysis times. The main motivation of our study was to develop an accurate and user-friendly tool that could be used to analyze large WGS datasets, such as the cancer genome atlas (TCGA) datasets. In our approach, the RD and BAF signals are smoothed, and double sliding windows subsequently are used to detect breakpoints, which makes our approach fast and straightforward. Because the breakpoint detection is performed simultaneously at different scales, it allows accurate detection. Our tool, Segmentum, is freely available under MIT license at: https://github.com/eafyounian/Segmentum (Additional file 2 contains the software code. For the lates version of the software code please visit the project’s online repository).

## Implementation

### Pipeline

**Fig. 1:**
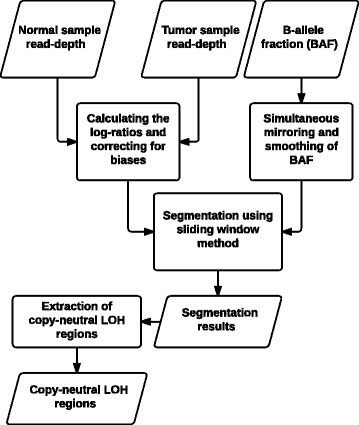
Segmentum pipeline. Normal and tumor RDs are used to calculate RD log-ratios. RD log-ratios are then corrected for biases. BAF data are simultaneously mirrored and smoothed. Using RD log-ratios and BAF, the genome is segmented with a double sliding window method. Segmentation results are used to identify cnLOH regions in the genome (see the following sections for more details on each step)

Segmentum was developed and written in the Python programming language (version 3) and requires the SciPy library to be installed (If the user wishes to use the ‘plot’ sub-command to inform parameter value selection, matplotlib library is also required). Segmentum employs SAMtools to extract RD and heterozygous SNPs BAF data from BAM files containing WGS data. These constitute the inputs required by Segmentum to perform copy number analysis. Figure 1 illustrates the Segmentum pipeline. Each step is explained in more detail in the following sections.

### RD extraction and BAF calculation for heterozygous SNPs

To extract the RD from the BAM files, the genome is divided into bins of user-defined length (2 kbp by default) and the number of reads overlapping each bin is counted to determine the RD at each bin. To calculate the BAF values, heterozygous SNPs in the normal sample are identified at known SNP sites in the human genome (based on SNP annotations such as those produced by the 1000 Genomes project). Next, the number of reference and alternative alleles at each heterozygous SNP position is extracted from the tumor sample and the BAF for the *i* ^*th*^ heterozygous SNP is calculated using the following equation:

$$ BA{F}_i = \frac{al{ t}_i}{al{ t}_i + re{f}_i} $$

where *alt*_*i*_ and *ref*_*i*_ refer to the alternative and reference allele, respectively, of the *i* ^*th*^ heterozygous SNP.

It should be noted that by default reads with mapping quality score 10 are filtered out before RD extraction and BAF calculation in order to address the challenges raised by reads not mapping to a unique region in the genome (the read filtration criterion based on the mapping quality score is a parameter to Segmentum and can be set by the user).

### Log-ratio calculation

The RD log-ratio is calculated using the following equation:

$$ log{r}_i = l o{g}_2\left(\ \frac{tR{ D}_i}{nR{ D}_i}\ \right) $$

where *logr*_*i*_ is the log-ratio of the *i* ^*th*^ genomic window and *tRD*_*i*_ and *nRD*_*i*_ are RDs extracted from the *i* ^*th*^ genomic window of a specific size (determined by user; default is 2 kbp) for the tumor and normal samples, respectively.

Differences in the total number of aligned reads in the normal and tumor samples may bias the estimation of the RD log-ratios. The correction was performed by finding the mode of log-ratio values for each chromosome and subtracting the median of all of the modes from each log-ratio value. It should be noted that median, in the correction step, is robust to the changes in one mode. For instance, one chromosomal arm having a copy number change has no effect on the correction since it only affects one of the chromosomal modes.

### Mirroring and smoothing of the BAF values

The BAF of a heterozygous SNP has an expected value of 0.5 in normal diploid cells. In the presence of somatic copy number alterations, the BAF can diverge from 0.5 if the relative abundance of the two alleles changes. To make smoothing and segmentation of BAF data possible, the BAF values must be mirrored about the 0.5 axis so that the B allele fraction always represents the allele fraction of the dominant allele. Without this mirroring step, the BAF values will be symmetric about the BAF = 0.5 axis and smoothing will underestimate the absolute divergence from 0.5 [14]. In this study, a median filter is used for smoothing the BAF data. Simultaneous mirroring and smoothing is implemented using the following equation:

$$ c B A{F}_i = H\ast \left|0.5-{M}_9\left( BA{F}_i\right)\right|+\left(1- H\right)\ast {M}_9\left(\left|0.5- BA{F}_i\right|\right). $$

where *BAF*_*i*_ is the BAF value for the *i* ^*th*^ heterozygous SNP, *cBAF*_*i*_ is the simultaneously mirrored and smoothed *BAF*_*i*_, *H* is a heterozygosity measurement calculated with the following equation: *H* = 1 − 2 ∗ |0.5 − *x*|, and *M*_9_ refers to applying a median filter to 9 SNPs in the vicinity of and including the *i* ^*th*^ SNP.

### Segmentation using a double sliding window approach

To detect changes in the RD log-ratio and BAF signals, two non-overlapping, fixed-sized windows (determined by the user) are slid over the RD log-ratio and BAF values and a compound score (*S*) is calculated for each of the adjacent two windows. If the compound score is greater than 1, a change is detected and a breakpoint is placed at the place where the two windows touch each other. The compound score is calculated using the following equation:

$$ S = \frac{\left|\ \overline{log{ r}_{wi{ n}_i}} - \overline{log{ r}_{wi{ n}_{i+1}}}\ \right|{}^2}{\tau_{log r}}+\frac{\left|\overline{cBA{ F}_{wi{ n}_i}} - \overline{cBA{ F}_{wi{ n}_{i+1}}}\ \right|{}^2}{\tau_{BAF}} $$

where $\overline{log{ r}_{wi{ n}_i}}$ is the mean of the RD log-ratio values in the *i* ^*th*^ window, $\overline{cBA{ F}_{wi{ n}_i}}$ is the mean of the mirrored and smoothed BAF values in the *i* ^*th*^ window, *τ*_*logr*_ and *τ*_*BAF*_ are thresholds for the absolute mean difference in the RD log-ratios and the absolute mean difference in the BAF values in the two adjacent windows, respectively.

It is possible that some breakpoints will not be detected by a single pass of a double sliding window due to a given window size. Thus, to increase the sensitivity, Segmentum analyzes the signals for the detection of breakpoints multiple times with different window-sizes and thresholds. Each new window is 1.5 times larger than the previous one. The increase in the window size decreases the detection thresholds. This is due to the fact that increasing the window size increases the sample size (assuming sampling from normal distribution with *N*(*μ*, *σ* ^2^)) and consequently decreases the standard deviation of the mean (mean having probability distribution of $$ N\left(\mu, \kern0.5em \raisebox{1ex}{${\sigma}^2$}\!\left/ \!\raisebox{-1ex}{$ n$}\right.\right) $$). The new standard deviation of the mean when window size is increased 1.5 times is $$ \frac{1}{\sqrt[2]{1.5}} $$ times the old standard deviation. Let *τ* = *ασ* where *τ* is the threshold and *α* is a scalar and *σ* is the standard deviation. It follows that:

$$ {\tau}_{new} = \alpha .{\sigma}_{new} = \frac{1}{\sqrt[2]{1.5}}.\ \alpha .{\sigma}_{old} = \frac{1}{\sqrt[2]{1.5}}.\ {\tau}_{old} $$

Thus both the *τ*_*logr*_ and *τ*_*BAF*_ thresholds are updated using the following equation:

$$ {\tau}_{new} = \frac{1}{\sqrt[2]{1.5}} \ast {\tau}_{old} $$

The process of increasing the window-size is continued as long as the updated thresholds are greater than the thresholds for the merging two consecutive segments (see below). After detecting all the breakpoints, a consensus list of breakpoints is created by accepting all of the breakpoints detected by the first pass of the double sliding window and adding the breakpoints detected from the larger windows to the list only if the breakpoint is not in the vicinity of an existing breakpoint in the list (i.e., |*cp*_*current*_ − *cp*_*existing*_| > *window size*, where *cp* is a detected breakpoint). Consensus breakpoints are used to create the segments. Two consecutive breakpoints constitute a segment. For each segment, the average RD log-ratio and average mirrored and smoothed BAF is calculated. Two consecutive segments are merged if the following conditions are met:

$$ \left|\ \overline{log{ r}_{se{ g}_i}} - \overline{log{ r}_{se{ g}_{i+1}}}\kern0.5em \right|<{\tau}_{merg{ e}_{log r}}\kern1em  and\kern1em \left|\ \overline{cBA{ F}_{se{ g}_i}} - \overline{cBA{ F}_{se{ g}_{i+1}}}\ \right| < {\tau}_{merg{ e}_{BAF}} $$

where $\overline{log{ r}_{se{ g}_i}}$ is the mean RD log-ratio of the *i* ^*th*^ segment, $\overline{cBA{ F}_{se{ g}_i}}$ is the mean mirrored and smoothed BAF of the *i* ^*th*^ segment, and ${\tau}_{merg{ e}_{logr}}$ and ${\tau}_{merg{ e}_{BAF}}$ (determined by user) are the RD log-ratio and BAF merging thresholds, respectively.

### Detection of cnLOH events within a single sample

A segment is considered to be a cnLOH segment if the following conditions are met:

$$ \left|\ \overline{log{ r}_{se{ g}_i}}\ \right| < {\tau}_{cnLO{ H}_{log r}}\kern1.25em  and\kern1.5em \left(0.5 - \kern0.5em \overline{cBA{ F}_{se{ g}_i}}\right) < {\tau}_{cnLO{ H}_{BAF}} $$

where $\overline{log{ r}_{se{ g}_i}}$ is the mean RD log-ratio of the *i* ^*th*^ segment, $\overline{cBA{ F}_{se{ g}_i}}$ is the mean mirrored and smoothed BAF of the *i* ^*th*^ segment, ${\tau}_{cnLO{ H}_{logr}}$ and ${\tau}_{cnLO{ H}_{BAF}}$ (determined by the user) are thresholds for calling a cnLOH segment.

### Detection of recurrent cnLOH regions across multiple samples

To find genomic regions with recurrent cnLOH events, all cnLOH regions for individual samples are identified following the procedure described earlier. Then, the number of occurrences of a cnLOH event for a specific region across multiple samples is counted using an interval tree data structure (Additional file 1: Figure S1).

### Simulator

To evaluate Segmentum in terms of segmentation accuracy, a simulator capable of simulating whole-genome RD for both normal and tumor samples and BAF based on events such as deletions, amplifications and cnLOH was developed. The simulator receives a normal sample RD data and outputs 4 sets of data including the simulated normal and tumor RD, BAF data and a ground truth. First, the simulator learns the distribution of the RD data from the provided normal sample by simply counting the number of times two consecutive RD values (e.g., 368 and 299) occur together throughout the genome (Additional file 1: Figure S2). The learned distribution also accounts for the inherent noise in the RD data. Next, inverse transform sampling (Smirnov transform) is used to generate RD values for each position in the genome based on the learned distribution. Then, noise is removed using a median filter. A normal RD is constructed by adding independent Poisson noise to the simulated RD data. To construct the tumor RD, two copy number tracks (because autosomal chromosomes come in maternal and paternal pairs) harboring random SCNAs are constructed. The tumor sample RD is calculated using the copy number tracks, the simulated normal sample RD and the normal sample contamination (i.e., a parameter determined by user). To construct the BAF data, heterozygous SNPs are initially randomly distributed across the genome (1 heterozygous SNP per 1.5 Kbp). The number of B-alleles at a heterozygous SNP is calculated using a binomial distribution with the parameters *n* (total number of reads at heterozygous SNP position) and *p* (probability that a read is coming from the B-allele). *n* is extracted from the simulated normal RD at heterozygous SNP positions. *p* is calculated using the two constructed copy number tracks and the normal sample contamination. Once the number of B-alleles is calculated, it is used to calculate the BAF values (Additional file 1: Figures S3 and S4 for the simulator pipeline and the simulated data visualized in the integrative genomics viewer (IGV) [15], respectively).

## Results

### Segmentum segmentation accuracy for the simulated data

**Fig. 2:**
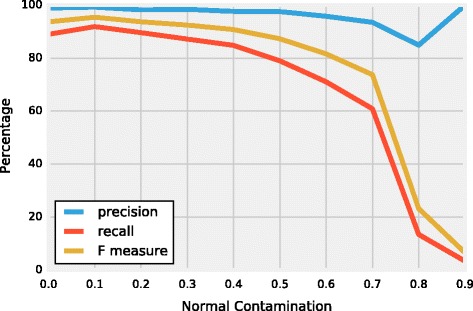
Segmentation accuracy of Segmentum for simulated data with different degrees of normal contamination. Estimated precision, recall, and F-measure values for simulated data at different normal contamination levels (Additional file 1, Derivation of the precision, recall, and F-measure of the simulated data)

Using the simulator (see the ‘Simulator’ section for more details), RD data for both normal and tumor samples and BAF values for heterozygous SNPs from the tumor sample as well as a ground truth were simulated with different percentages of normal contamination (an example set of simulated data is available at Segmentum’s online repository. See the ‘Availability and requirements’ section for the link to the repository). The simulated data were analyzed by Segmentum. The segmentation results were evaluated against the ground truth. The precision, recall, and the F-measure values were calculated based on this evaluation (Fig. 2 and Additional file 1 for the definitions of precision, recall, and F-measure).

### Segmentum segmentation accuracy for real data compared to other tools

**Fig. 3:**
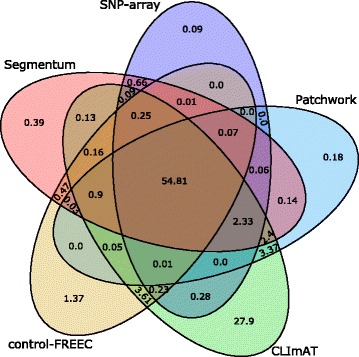
Comparison of the SCNA results with different tools and the SNP array (ground truth). Venn diagram values (averaged over ten TCGA LGG samples) represent the percentage of overlap among the SCNA calls

To assess segmentation accuracy of Segmentum for real data, paired tumor/normal whole genome sequencing samples (30x < coverage < 100x) from 10 individuals diagnosed with low-grade glioma (LGG) were downloaded from the TCGA dataset and used as is. Furthermore, segmentation results from SNP-array data (level 3 data) (completed by TCGA using an Affymetrix Genome-wide human SNP array 6.0) was used as ground truth (Additional file 1: Table S3). Segmentum’s results were evaluated against Control-FREEC, Patchwork, and CLImAT as competing tools. To evaluate the segmentation accuracy, the genome was broken into 100 bp. blocks (excluding all blocks in centromeres and sex chromosomes). Using block annotations from different tools, genome-wide proportions of the blocks annotated as SCNA by different combinations of tools were calculated and the results were illustrated by a Venn diagram (Fig. 3).

**Fig. 4:**
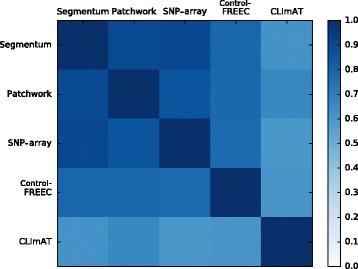
Pairwise JSI scores averaged over ten TCGA LGG samples. JSI scores range between 0 and 1, where 0 means no similarity and 1 represents identical results between two tools

Additionally, to measure the pairwise degree of similarity of the segmentation results between two tools, the *Jaccard similarity index* (JSI) was calculated for all of the pairs using the following equation:

$$ J S I = \frac{\left|\cap pair\right|}{\ \left|\cup pair\right|\ } $$

where | ∩ *pair*| and | ∪ *pair*| are the cardinalities of intersection and union, respectively. Intersection and union values were extracted from the Venn diagrams. Figure 4 represents a heat map of the JSI values for each pair of tools averaged over 10 TCGA LGG samples. According to the heat map, on average, Segmentum produces the most similar results to the SNP array segmentation results with a JSI score of 0.9, followed by Patchwork with a JSI score of 0.86.

Similar evaluations using low coverage data (6x average coverage) are shown in Additional file 1: Figures S5 and S6. The low coverage data is comprised of the paired tumor/normal whole genome sequencing samples of 10 individuals diagnosed with prostate adenocarcinoma (PRAD). With regard to the low coverage data, Patchwork produces the most similar results to the SNP array segmentation results with a JSI score of 0.93, followed by Segmentum with a JSI score of 0.88. Additional file 1: Table S4 contains the names of the 10 TCGA PRAD samples (Additional file 1: Tables S6-S10 represent the parameter values used for running the competing tools. Additional file 1: Segmentum’s parameter value selection section provides guidance on selecting parameter values for Segmentum. Additional file 1: Figure S9 represents an example plot made by Segmentum’s 'plot' sub-command that can be used to guide the parameter value selection).

### Segmentum segmentation accuracy for the subsampled real data

**Fig. 5:**
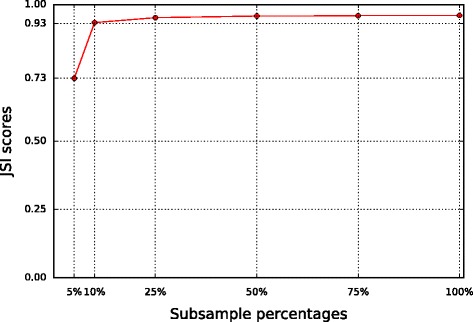
Pairwise JSI scores (Segmentum vs. SNP array as ground truth) for different subsamples. JSI scores range between 0 and 1, where 0 means no similarity and 1 represents identical results between two tools

To assess the segmentation accuracy of Segmentum for real data with respect to sample’s coverage, we subsampled one of the LGG samples (i.e. TCGA-CS-5395) at different subsampling fractions (i.e. 75%, 50%, 25%, 10%, and 5%) using Samtools (version 1.3.1). We analyzed each subsample by Segmentum and benchmarked it against ground truth in the same manner as explained earlier. Figure 5 represents the JSI scores for each subsample (Additional file 1: Figure S7 shows the average coverage of the subsamples for normal and tumor pairs). It can be seen that Segmentum reaches high accuracies even with low coverage data. For instance, the accuracy for the 10%-fraction subsample was 93.4% (where the average coverage for tumor and normal subsamples were 3 and 4 respectively).

It should be noted that as the coverage decreases the number of identified heterozygous SNPs decreases (Additional file 1: Figure S8). For instance, for the 10%-fraction subsample only 1997 heterozygous SNPs were identified from the entire genome (in contrast to the original sample where the number of identified heterozygous SNPs was more than 3 million SNPs). Even though Segmentum is shown to work with low coverage data, one should note the implications of low amounts of detected heterozygous SNPs on the reliable detection of cnLOH events.

### Time usage evaluation

All of the computations were completed on the same UNIX server. Table 1 shows the average time required by each tool to perform the analysis for 10 TCGA LGG samples (30x < coverage < 100x). Based on the results, on average, CLImAT appears to be the fastest, followed by Segmentum, Patchwork, and Control-FREEC. It should be noted that to assign the allele-specific copy number to genomic segments, Patchwork requires users to determine some parameter values by interpreting plots produced by the tool, and this interpretation time is not included here. Additionally, the time required to create the pileup files used by Patchwork and Control-FREEC is different due to the use of different parameter values in SAMtools. It should be noted that time required for making pileup files can be decreased by parallelizing the process on machines with multiple cores or on computer clusters (e.g. by assigning one core to each chromosome). Similarly, BAF calculation for Segmentum can be parallelized. However, since this is not a core feature of the benchmarked tools and not all tools support parallelization, to be fair, only the required linear time is reported here. A similar time usage evaluation, using low coverage data (average coverage 6x), is shown in Additional file 1: Table S2. With regard to the low coverage data, Segmentum comes second after CLImAT in terms of analysis time, which is consistent with the results from the high coverage data.

**Table 1:** Average tool analysis time for high coverage data (30x < coverage < 100x)

Tool	Average preparation time	Average analysis time
Segmentum	- 10 h 34 min for extracting RD from normal or tumor BAM file - 4 h 25 min for calculating BAF values	- 1 min 45 s
Patchwork	- 29 h 37 min for creating pileups from normal or tumor BAM file	- 3 h 56 min
Control-FREEC	- 33 h 28 min for creating pileups from normal or tumor BAM file	- 7 h 11 min
CLImAT	- 2 h 12 min for extracting RD	- 29 min

### Recurrent cnLOH detection case study

**Fig. 6:**
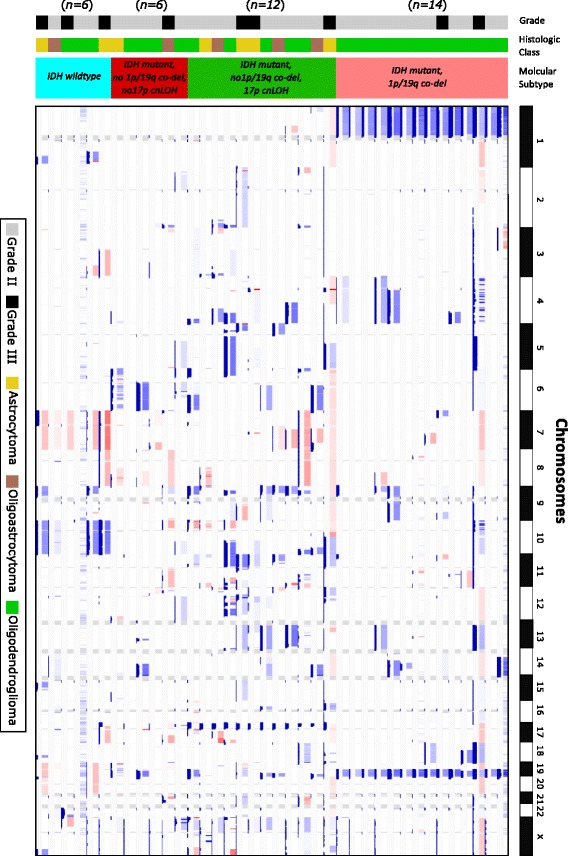
SCNA landscape in grade II and III gliomas. WHO-grade, histological class, and molecular subtype classification are shown by color as indicated. The thirty-eight samples are divided into 4 distinct subtypes based on the occurrence of a mutation in *IDH1/2*, co-deletion of chromosomes 1p and 19q and the presence of 17p cnLOH. Deletions and amplifications are visualized by boxes with different shades of blue and red, respectively. White regions are either normal or cnLOH regions. The bar charts below each box represent the mirrored and smoothed BAF values. Large mirrored and smoothed BAF values (close to 0.5) point to heterozygous SNP allelic imbalance. In the second subtype (from the top), at chromosome 17p, recurring cnLOH is apparent where the bar charts point to large mirrored and smoothed BAF values, though no deletion or amplification is detected at that region (Additional file 1: Table S5 for TCGA LGG sample barcode names)

In a study of lower grade gliomas (LGGs), i.e., grade II and III gliomas, Suzuki et al. [4] characterized the mutational landscape of these glioma types by dividing them into 3 distinct subtypes based on their distinct sets of mutations and clinical behaviors. These subtypes are distinguished with the following criteria: (1) mutation in *IDH1/2* accompanied by co-deletion of chromosomes 1p and 19q (subtype I), (2) mutation in *IDH1/2* without co-deletion of chromosomes 1p and 19q (subtype II), and (3) *IDH1/2* wild type (subtype III). Of interest to our study was the recurrence of cnLOH events in chromosome 17p in subtype II [4]. To show the ability of Segmentum to detect such aberrations from large datasets, 38 paired-end WGS samples from the TCGA dataset (30x < coverage < 100x)) for patients diagnosed with LGG were downloaded and analyzed by Segmentum. We were able to distinguish all three subtypes as characterized in [4], including the recurrence of cnLOH in subtype II at chromosome 17p. We also identified a fourth subtype with a mutation in *IDH1/2* without co-deletion of chromosomes 1p and 19q and no cnLOH at 17p (Fig. 6).

## Discussion

By comparing the simulated (Fig. 2) and real data (Figs. 3, 4 and 5 and Additional file 1: Figures S5 and S6), we can conclude that Segmentum can recover true copy number aberrations with high accuracy even when the coverage is as low as ~4 reads (Fig. 5, Additional file 1: Figure S7). On average, Segmentum produces results that are the most concordant with the copy number aberrations identified from the SNP array data (i.e. ~90% of concordance) (Fig. 4). As shown in Table 1, our tool is more than twice as fast as the second best performing tool in terms of accuracy. Segmentum is also the second fastest tool after CLImAT compared to the other tools evaluated in this study (Table 1). However, CLImAT ranks last in terms of accuracy (Fig. 4). One explanation for the speed of CLImAT is that it computes the BAF values for a subset of known SNPs (~13.7 million SNPs that are retrieved from the dbSNP database [16]). In contrast, Segmentum, computes the BAF values for heterozygous SNPs determined from the 1000 Genomes project’s SNP list (~85 million SNPs) [17]. The other reason for the speed of CLImAT might be that it does not require a normal sample for analysis.

As the normal contamination in the simulated data increases, the number of false negatives increases and the recall rate decreases (Fig. 2). However, within the ranges of realistic amounts of normal contamination (i.e. ~30% to 40%), Segmentum performs consistently well.

Segmentum is able to report recurrent cnLOH regions across multiple cancer genome samples; a characteristic of cancer genomes that has been neglected until recently [4]. By applying Segmentum to TCGA data, we were able to recover recurrent cnLOH events from low-grade glioma samples that were reported earlier by SNP array-based data analysis. It is worth mentioning that Segmentum can work in two modes, i.e., with or without BAF value. In the case where BAF values are not used, Segmentum cannot detect regions with cnLOH. Furthermore, Segmentum is capable of reliably segmenting the cancer genome using both high (Figs. 3 and 4) and low (Fig. 5 and Additional file 1: Figures S5 and S6) sequence coverage data. However, with the low sequence coverage data, the estimated BAF values for the heterozygous SNPs will be less reliable. This is also reflected in Additional file 1: Figure S8, where it is shown that the number of detected heterozygous SNPs drop as the average coverage decreases. The implications of low amounts of detected heterozygous SNPs on the reliable detection of cnLOH events should not be overlooked.

Even though we have shown that Segmentum is highly accurate at recovering the true copy number, other tools in this study do more than just segmenting the genome. For instance, CLImAT and Patchwork are capable of estimating tumor ploidy and tumor purity and consequently, reporting the integral copy numbers for each segment. Patchwork and Control-FREEC are also capable of reporting the genotype of each segment and CLImAT reports the genotype for each SNP within each segment. This is in contrast to Segmentum that only reports the mean RD log-ratio and BAF value of each segment. However, tools such as ABSOLUTE [18] or THetA [19] can be used to estimate tumor impurity and ploidy from Segmentum’s segmentation result, meaning that Segmentum can be used as part of a larger tumor evolution analysis pipeline. Finally, a strength of our tool is its minimum dependence on third party tools, with the exception of SAMtools, for calculating the RD and BAF.

## Conclusions

We have developed Segmentum as a tool for the identification of SCNAs, including cnLOH in tumor samples, using WGS data. We have shown that Segmentum is accurate and fast with regards to other state-of-the-art tools, making it suitable for analyzing cohorts with a large number of samples, such as TCGA cohorts.

## Availability and requirements

**Project name:** Segmentum

**Project homepage:** https://github.com/eafyounian/Segmentum

**Operating system(s):** Linux

**Programming language:** Python

**Other requirements:** SciPy, Samtools, and matplotlib if the ‘plot’ sub-command is used.

**License:** MIT license

**Any restrictions to use by non-academics:** None

## Additional files

Additional file 1: This file contains supplementary information, tables and figures supporting the manuscript. **Figure S1.** Detection of regions harboring recurrent cnLOH across multiple samples. **Figure S2.** Read depth spatial correlation. **Figure S3.** Simulator pipeline. **Figure S4.** Simulated data visualized in Integrative Genomics Viewer (IGV). **Figure S5.** Comparison of SCNA results from different tools and SNP array (ground truth) for low sequence coverage data. **Figure S6.** Pairwise JSI scores for low sequence coverage data (averaged of 10 TCGA PRAD samples) **Figure S7.** Subsample average coverages in the subsampling evaluation. **Figure S8** Detected number of heterozygous SNPs in different subsamples **Figure S9.** Copy number – B-allele fraction clusters. **Table S1.** List of SCNA tools using WGS data. **Table S2.** Average tool analysis time for low sequence coverage data (average coverage 6x). **Table S3** TCGA LGG sample barcode names and the estimated sample purity by ABSOLUTE. **Table S4.** TCGA PRAD sample barcode names and the estimated sample purity by ABSOLUTE. **Table S5.** TCGA LGG sample barcode names categorized based on inferred subtype. **Table S6.** Parameter values for running DFExtract. **Table S7.** Parameter values for running CLImAT. **Table S8.** Parameter values for running Patchwork for 10 TCGA LGG samples. **Table S9.** Parameter values for running Patchwork for 10 TCGA PRAD samples. **Table S10.** Parameter values for running Control-FREEC. (DOCX 857 kb)

Additional file 2: Software code. This compressed file contains the software code (for the latest version of the software code please visit the project’s online repository). (ZIP 32 kb)

## Abbreviations

BAF: B-Allele fraction
BAM: Binary alignment map
CBS: circular binary segmentation
CGH: Array comparative genomic hybridization
cnLOH: Copy-neutral loss of heterozygosity
CNV: Copy number variation
dbSNP: Single nucleotide polymorphism database
DNA: Deoxyribonucleic acid
FISH: Fluorescent in situ hybridization
HMM: Hidden Markov model
HTS: High throughput sequencing
IGV: Integrative genomics viewer
JSI: Jaccard similarity index
LASSO: Least absolute shrinkage eStimatOr
LGG: Low grade glioma
LOH: Loss of heterozygosity
MAE: Monoallelic expression
PRAD: PRostate ADenocarcinoma
RD: Read-depth
SAM: Sequence alignment/map
SCNA: Somatic copy number alteration
SNP: Single nucleotide polymorphism
TCGA: The cancer genome atlas
WES: Whole exome sequencing
WGS: Whole genome sequencing
