# Supplementary material for: Segmentum: a tool for copy number analysis of cancer genomes
Source: BMC Bioinformatics. 2017 Apr 13;18:215. doi: 10.1186/s12859-017-1626-8 (PMC5390478; doi:10.1186/s12859-017-1626-8)
Supplement: Supplementary file 1 — This file contains supplementary information, tables and figures supporting the manuscript. Figure S1. Detection of regions harboring recurrent cnLOH across multiple samples. Figure S2. Read depth spatial correlation. Figure S3. Simulator pipeline. Figure S4. Simulated data visualized in Integrative Genomics Viewer (IGV). Figure S5. Comparison of SCNA results from different tools and SNP array (ground truth) for low sequence coverage data. Figure S6. Pairwise JSI scores for low sequence coverage data (averaged of 10 TCGA PRAD samples) Figure S7. Subsample average coverages in the subsampling evaluation. Figure S8 Detected number of heterozygous SNPs in different subsamples Figure S9. Copy number – B-allele fraction clusters. Table S1. List of SCNA tools using WGS data. Table S2. Average tool analysis time for low sequence coverage data (average coverage 6x). Table S3 TCGA LGG sample barcode names and the estimated sample purity by ABSOLUTE. Table S4. TCGA PRAD sample barcode names and the estimated sample purity by ABSOLUTE. Table S5. TCGA LGG sample barcode names categorized based on inferred subtype. Table S6. Parameter values for running DFExtract. Table S7. Parameter values for running CLImAT. Table S8. Parameter values for running Patchwork for 10 TCGA LGG samples. Table S9. Parameter values for running Patchwork for 10 TCGA PRAD samples. Table S10. Parameter values for running Control-FREEC. (DOCX 857 kb) [file 12859_2017_1626_MOESM1_ESM.docx]

Supplementary information

Segmentum: a tool for copy number analysis of cancer genomes

Ebrahim Afyounian^1^, Matti Annala^1^, Matti Nykter*^1^

^1^ Faculty of Medicine and Life Sciences and BioMediTech institute, University of Tampere, Tampere, Finland.

*corresponding author

Derivation of precision, recall and F-measure for simulated data

To calculate the precision, recall, and F-measure, a confusion matrix was populated by counting the true positives, false negatives and false positives. If a breakpoint from the segmentation results was within 10 kbp of a breakpoint in the ground truth, it was accepted as a true positive; otherwise it was a false positive. If there was no corresponding breakpoint in the results for a breakpoint in the ground truth, it was counted as a false negative. Precision, recall, and F-measure were calculated using following equations:

$$precision =\frac{TP}{TP+ FP}$$

$$recall = \frac{TP}{TP+FN}$$

$$F_{measure}=2*\frac{precision * recall}{precision + recall}$$

where $TP$ is the true positive count, $FP$ is the false positive count, and $FN$ is the false negative count.

For Figure 2 in the manuscript, Segmentum was run with simulated data with the following parameters: (1) window-size=11 kbp, (2) RD log-ratio threshold=0.7 and (3) BAF threshold=0.2.

Overview of tools

Control-FREEC

Control-FREEC (Control-FREE Copy number and allelic content caller) is a read-depth-based method for the detection of somatic copy number variations and LOH written in C++ programming language [1]. Control-FREEC constructs the copy number and BAF profiles using aligned reads and genomic position data of known SNPs (retrieved from dbSNP database). Subsequently, Control-FREEC normalizes, segments, and analyzes the constructed profiles to determine the copy number and allelic content. Control-FREEC uses equally sized, non-overlapping windows to compute the read-depth ratios for each window. The use of a control sample is optional. If a control sample is not available, Control-FREEC estimates a hypothetical read-depth for a given window using a polynomial function based on the window’s GC content. However, if a control sample is available, it can distinguish somatic from germline variants. To perform the segmentation, Control-FREEC determines the breakpoints using a Least Absolute Shrinkage eStimatOr (LASSO) regression. One feature of Control-FREEC is its ability to evaluate and correct for normal cell contamination, GC-content, and mapability biases while constructing the copy number profile of a tumor genome [1]. However, the user should determine the sample ploidy. If ploidy is not known, it is suggested to run the program several times with possible ploidy values and compare the results [2].

Patchwork

Patchwork is a read-depth-based method for performing allele-specific somatic copy number analysis [3]. Patchwork is written in the R programming language. Patchwork automatically calculates the average ploidy and purity of the tumor cells, and therefore does not require prior knowledge of the average ploidy (as opposed to Control-FREEC) or tumor cell content. Patchwork starts by taking aligned reads in BAM format. Then, it performs GC-normalization followed by a positional normalization. After normalization, Patchwork uses equally sized 10 kbp windows to compute the normalized read-depth for each window. These RDs are then used to segment the genome using a circular binary segmentation (CBS) algorithm where each segment is assigned an average normalized coverage. Using SAMtools, the single nucleotide variant data are extracted and informative heterozygous variants from a list of known SNPs (from the dbSNP database) are identified. Using these data, the allelic imbalance ratios are calculated and assigned to each segment. Subsequently, Patchwork visualizes the allelic imbalance ratio and normalized coverage for genomic segments from each chromosome. Using these visualizations, a user will be able to determine the parameters needed by Patchwork to assign allele-specific copy number to genomic segments.

CLImAT

CLImAT (CNA and LOH Assessment in Impure and Aneuploid Tumors), which was suggested by Yu et al. [4] and written in the C and Matlab programming languages, is a read-depth-based method for assessing the somatic copy number variation and LOH. CLImAT is capable of estimating tumor impurity and ploidy. Furthermore, CLImAT does not require the use of a control sample. CLImAT starts by taking aligned reads in BAM format and a file containing a list of all known SNPs (retrieved from dbSNP database). SAMtools is used to extract the read-depth data from the BAM file by counting the reads starting at a position within a 1000 base pair window centered at each of the known SNPs. Once the read-depth data are extracted, it is corrected for CG-content and mapability biases. Then, BAF data for each SNP is calculated and normalized (using quantile normalization) to eliminate allelic bias. Allelic bias refers to an issue where most aligners prefer to align reads to a reference allele than the alternative allele. Once the read-depth and BAF data are ready, CLImAT models them with an integrated HMM to infer the somatic copy number variation, LOH, tumor ploidy, tumor cell content and tumor genotype.

**Table S1.** List of SCNA tools using WGS data.

| **Name** | **Journal and year published** | **Input** | **Methods used** | **Need of control** | **Programming language** | **Requires BAF** | **Supported sequencing technology** |
| --- | --- | --- | --- | --- | --- | --- | --- |
| **SegSeq** | Nature Methods, 2009 | BED | Statistical testing, CBS | Yes | Matlab | No | Massively parallel sequencing |
| **readDepth** | PLOS ONE, 2011 | BED | LOESS regression, CBS | No | R | No | Massively parallel sequencing |
| **rSW-seq** | BMC Bioinformatics, 2010 | Read-depth | Smith-Waterman Algorithm | Yes | C | No | WGS, Single-end sequencing |
| **GENSENG** | Nucleic Acids Research, 2013 | Triplet of RD signal, GC content, Mapability | HMM, Negative binomial regression | No | C++ | No | WGS |
| **CLImAT** | Bioinformatics, 2014 | BAM,  GC content, Mapability | HMM | No | Matlab, C | Yes | WGS |
| **Control-FREEC** | Bioinformatics, 2012 | BAM, SAM, Pileup, SNP, Mapability | Lasso based | Optional | C++ | Yes | WGS, WES |
| **Patchwork** | Genome Biology, 2013 | BAM, Pileup | CBS | Optional | R | Yes | WGS |
| **SeqCNA** | BMC genomic, 2014 | SAM | LOESS | No | R | No | WGS |
| **TITAN** | Genome research, 2014 | Wig files outputted from HMMcopy | HMM | Yes | R | Yes | WGS |
| **FALCON** | Nucleic Acid Research 2014 | read counts for variant allele | bivariate mixed Binomial process | Yes | R | Yes | WGS |
| **Sequenza** | Annals of Oncology, 2014 | BAM, pileup | ‘Copy number’ R package | Yes | R, Python | Yes | WGS, WES |
| **FACETS** | Nucleic Acids Research, 2016 | BAM, snp-pileup | Non-parametric joint segmentation using Hotelling T^2^ statistic | Yes | R, Perl | Yes | WGS, WES, Targeted panel sequencing |


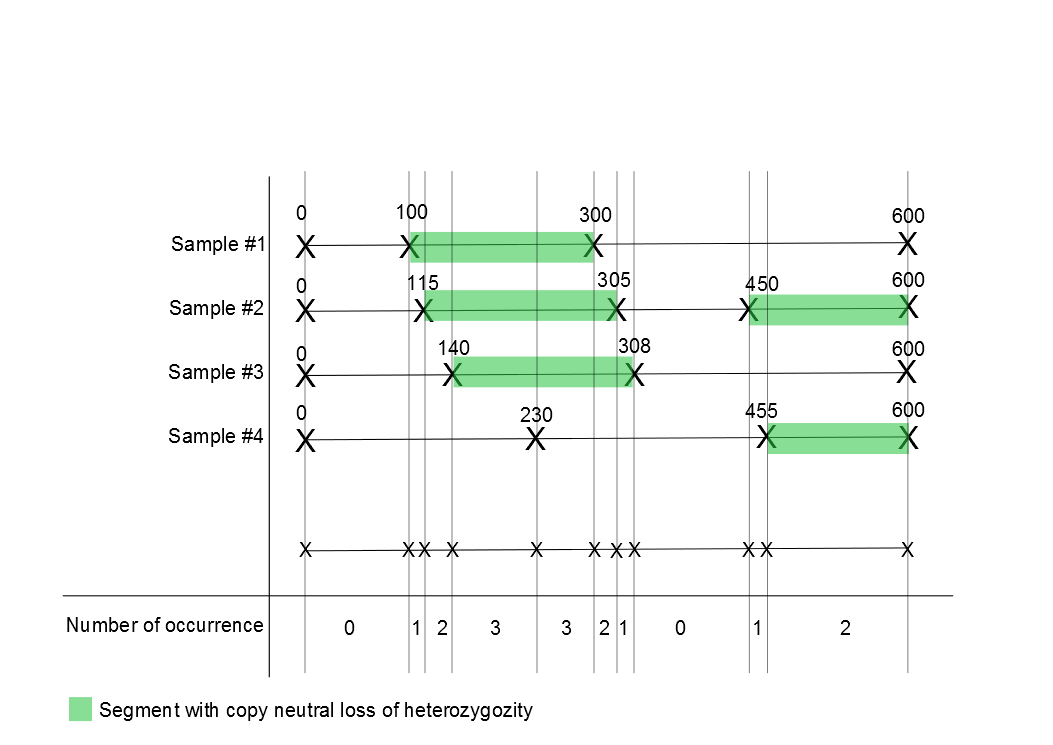


**Figure S1.** Detection of regions harboring recurrent cnLOH across multiple samples. A set of breakpoint positions from all of the samples is constructed. Each two consecutive breakpoint positions in the set constitute a segment. The number of times cnLOH occurs within that segment across all of the samples is counted and reported.


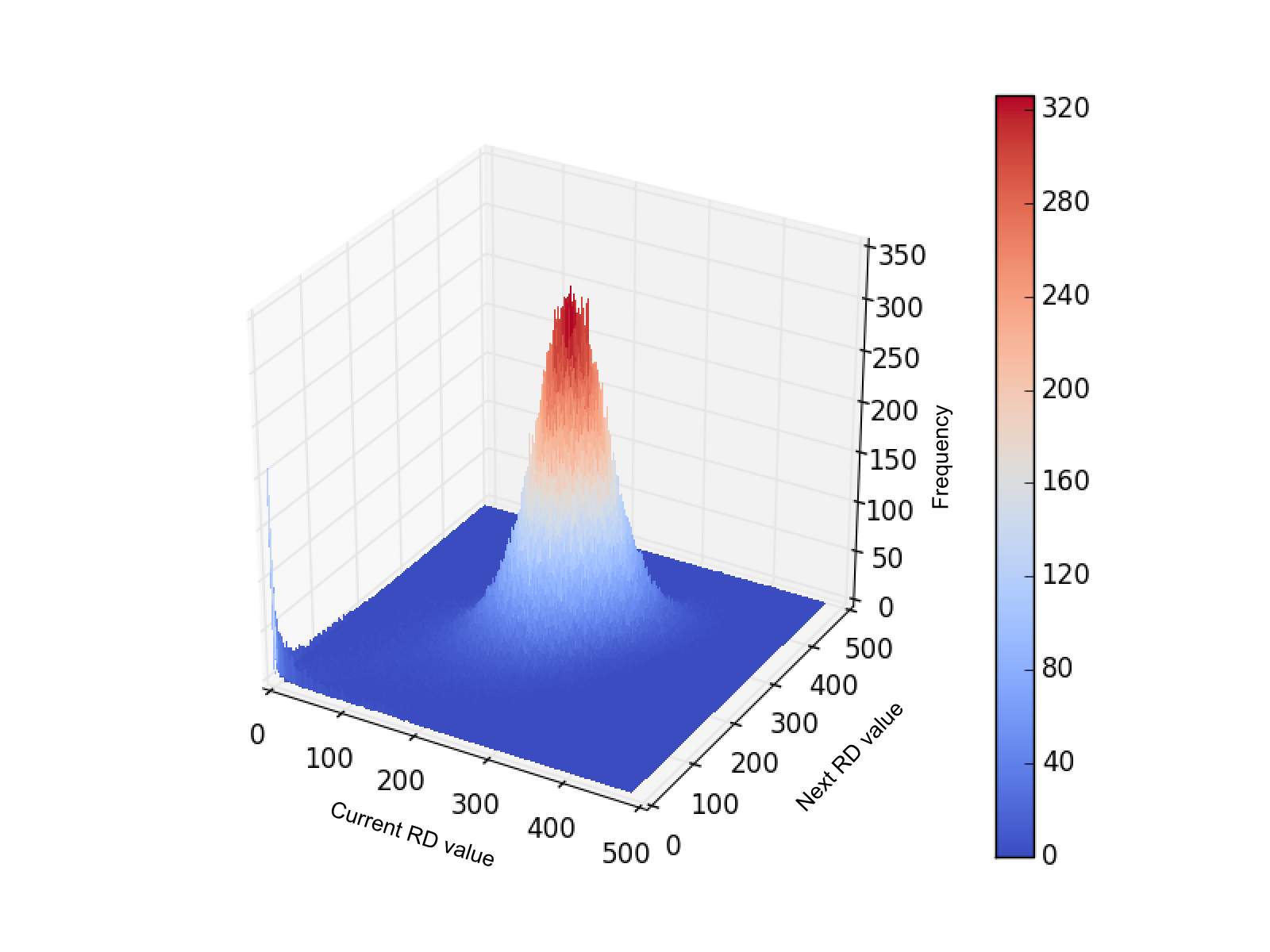


**Figure S2.** Read depth spatial correlation. This figure represents the occurrence frequency of two consecutive read depth (RD) values throughout the genome. RD values are calculated from a normal sample BAM file with an average coverage of 6x.


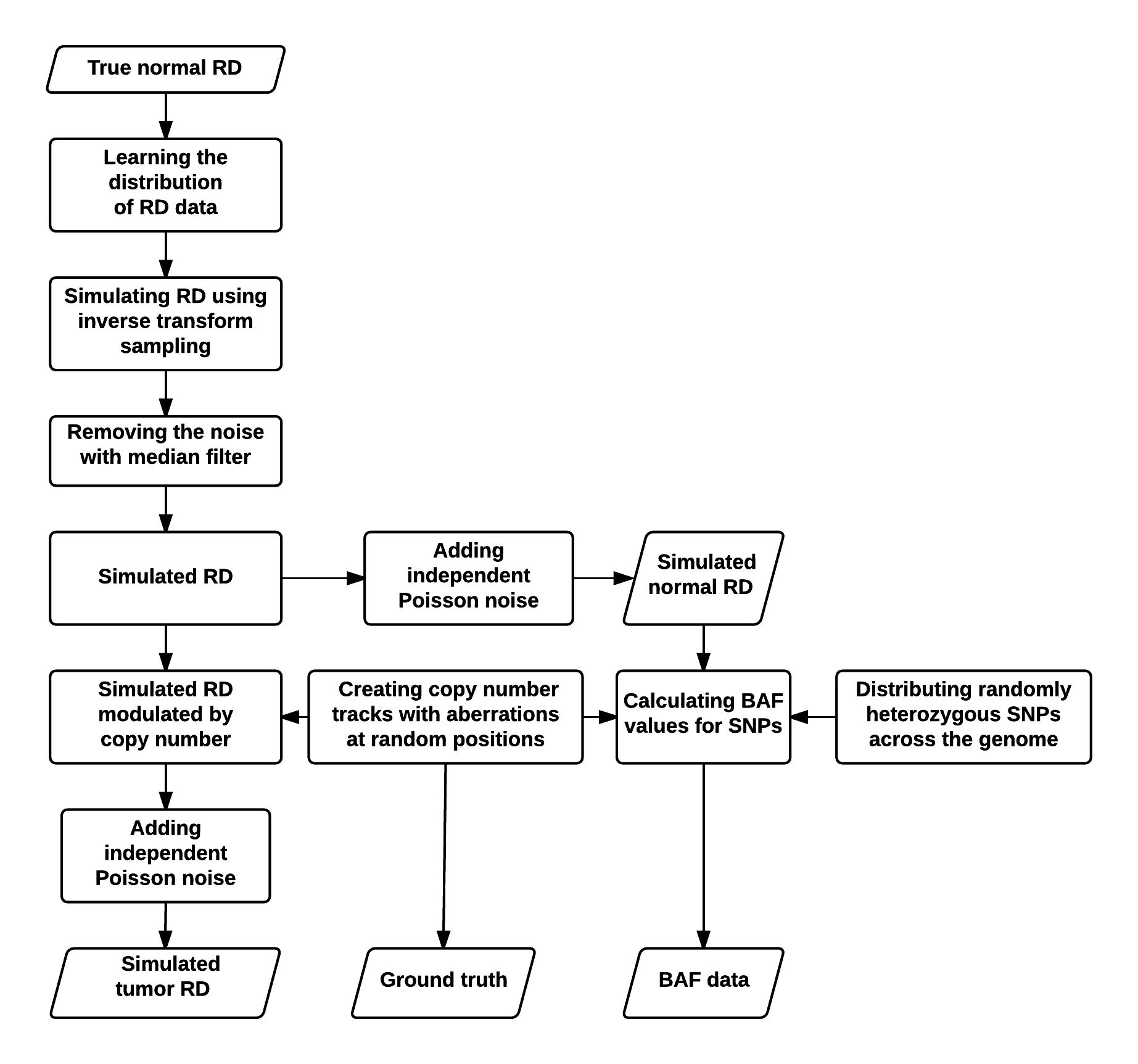


**Figure S3.** Simulator pipeline (see the main manuscript for description).


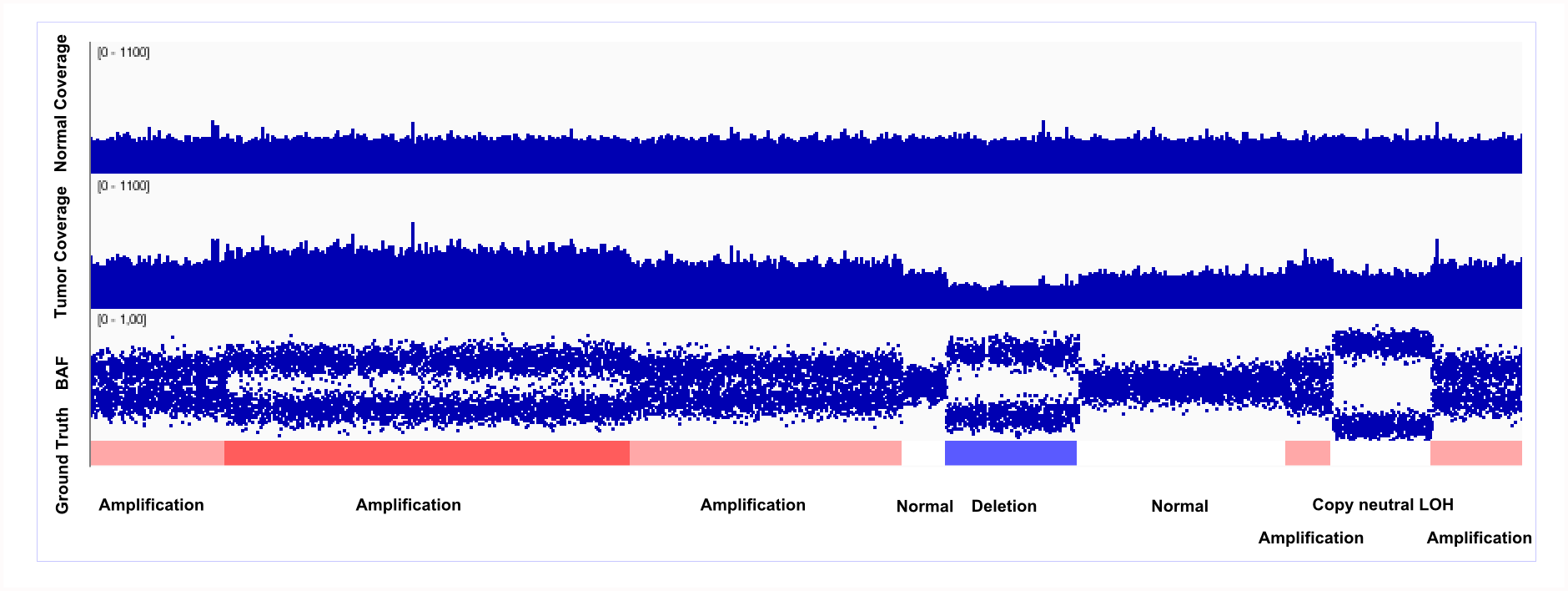


**Figure S4.** Simulated data visualized in Integrative Genomics Viewer (IGV). The first and second tracks represent the simulated normal and tumor RD values, respectively. The third track represents the simulated BAF at heterozygous SNPs. The fourth track represents the ground truth. Blue and red colored boxes represent the deleted and amplified segments, respectively. White boxes represent either normal or cnLOH segments.

Segmentation accuracy and time usage evaluation for low sequence coverage samples

To assess the segmentation accuracy of Segmentum for real data with low sequence coverage, paired tumor/normal whole genome sequencing samples (6x average coverage) from 10 individuals diagnosed with prostate adenocarcinoma (PRAD) from the TCGA dataset were downloaded and used as is. SNP-array data (level 3 data) segmentation results for the samples (completed by TCGA using an Affymetrix Genome-wide human SNP array 6.0) were used as the ground truth (Table S4 shows the sample barcode names). The same procedure described in the main manuscript was followed to evaluate the segmentation accuracy and time usage. Figures S5 and S6 and Table S2 show the results obtained for low sequence coverage data.


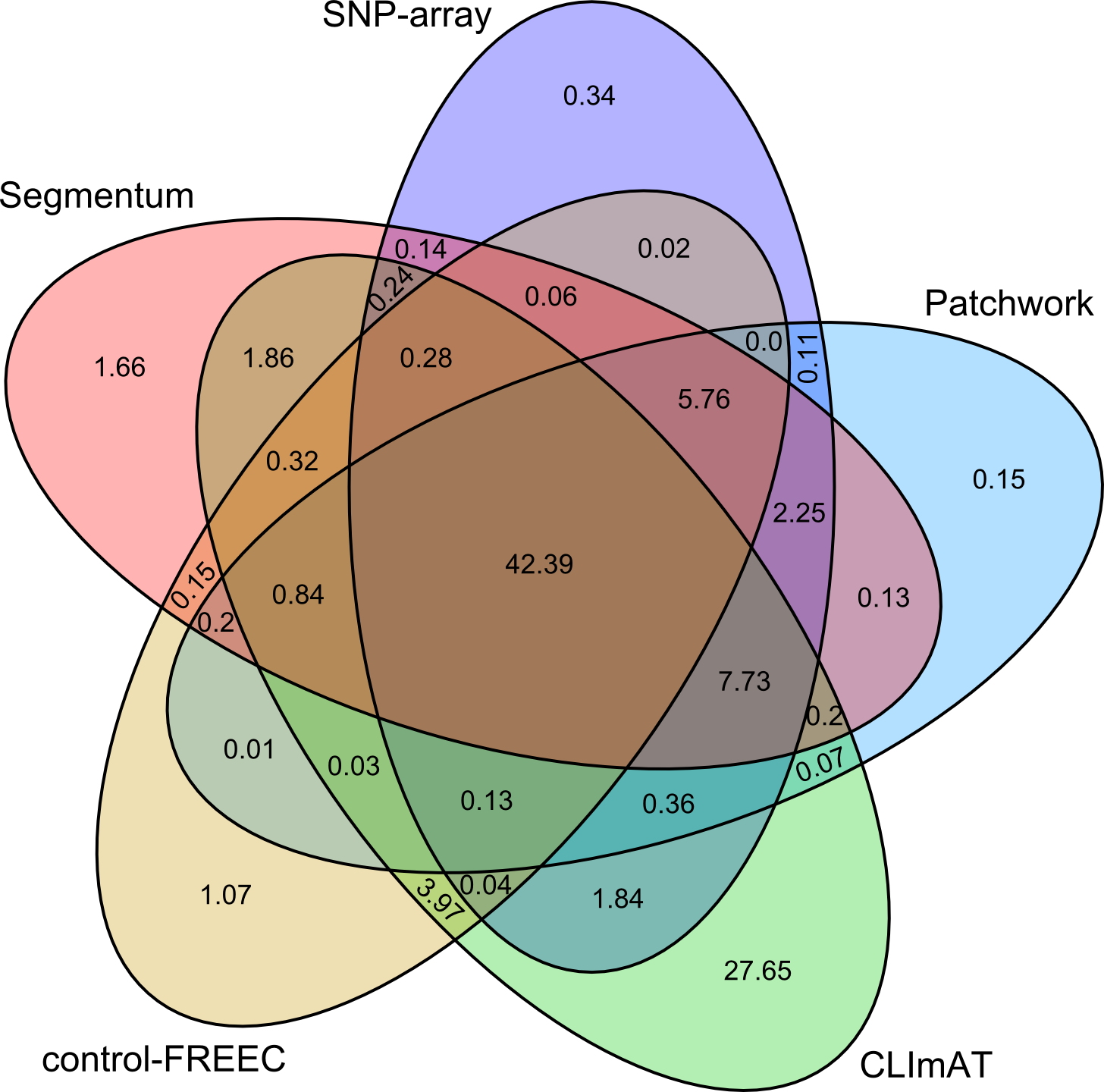


**Figure S5.** Comparison of SCNA results from different tools and SNP array (ground truth) for low sequence coverage data. Venn diagram values (averaged for 10 TCGA PRAD samples) represent the percentage of overlap among the SCNA calls.


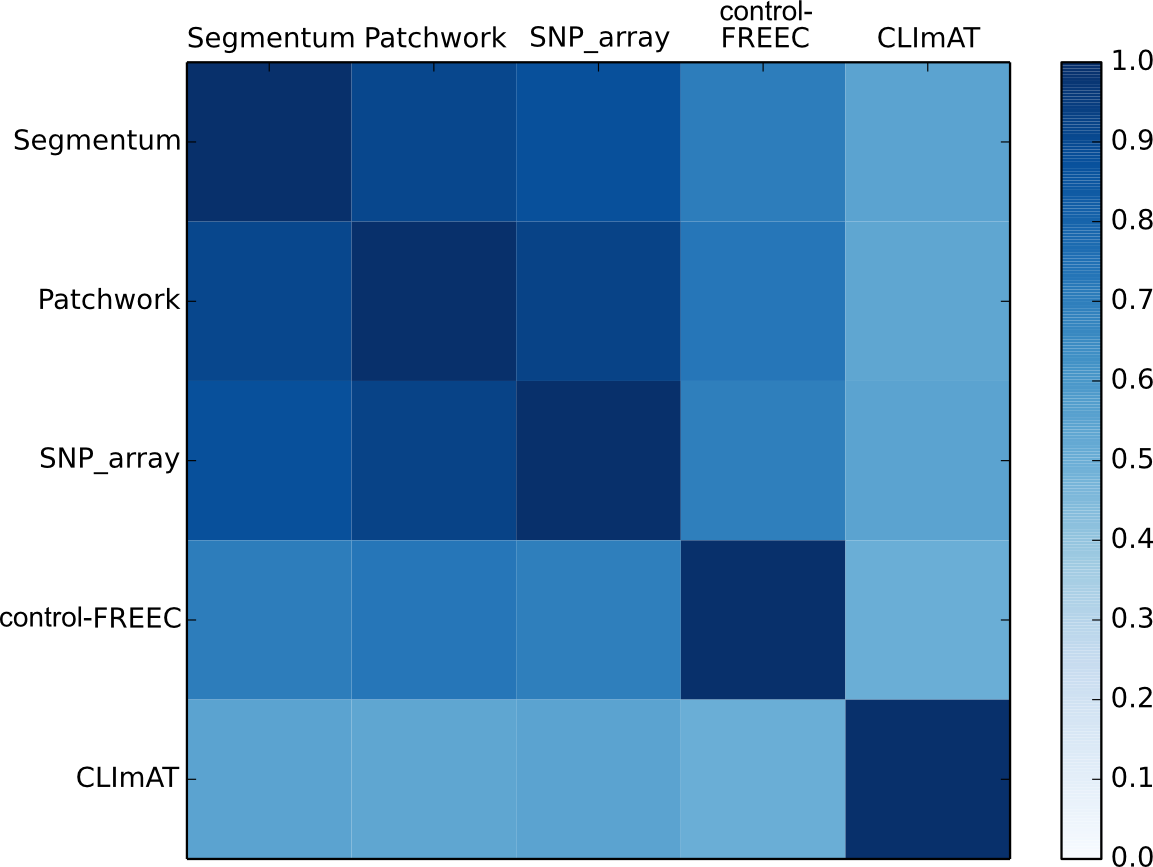


**Figure S6.** Pairwise JSI scores for low sequence coverage data (averaged of 10 TCGA PRAD samples). JSI scores range between 0 and 1, where 0 means no similarity and 1 represents identical results between two tools.

**Table S2.** Average tool analysis time for low sequence coverage data (average coverage 6x)

| **Tool** | **Average preparation time** | **Average analysis time** |
| --- | --- | --- |
| **Segmentum** | - 3 hours 50 minutes for extracting RD from normal or tumor BAM file  - 45 minutes for calculating BAF values | - 32 seconds |
| **Patchwork** | - 3 hours 50 minutes for creating pileups from normal or tumor BAM file | - 1 hour 7 minutes |
| **Control-FREEC** | - 5 hours 15 minutes for creating pileups from normal or tumor BAM file | - 3 hours 53 minutes |
| **CLImAT** | - 18 minutes for extracting RD | - 14 minutes |

##

| 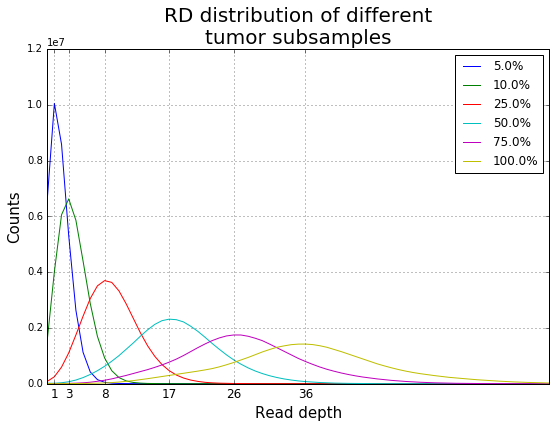 | 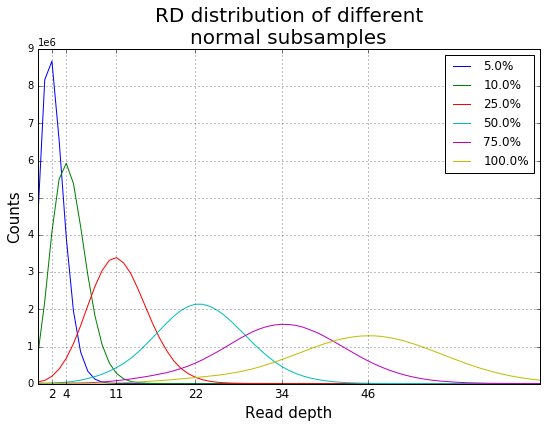 |
| --- | --- |

**Figure S7.** Subsample average coverages in the subsampling evaluation. Left panel represents the average coverages of subsamples of a tumor sample. Right panel represents the average coverages of subsamples of the paired normal sample.

**
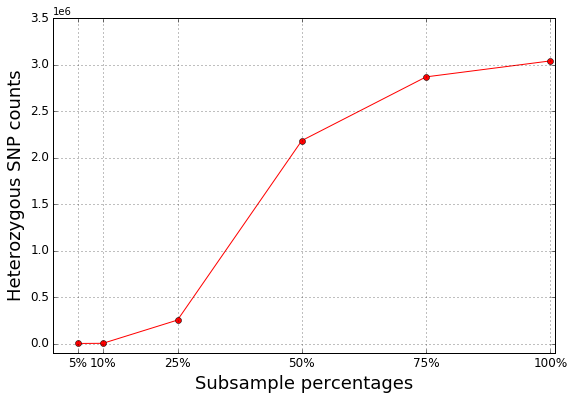
**

**Figure S8.** Detected number of heterozygous SNPs in different subsamples (using Segmentum default parameter values as indicated in the user interface).

Segmentum’s parameter value selection

The resulting plot from ‘plot’ sub-command in Segmentum guides the user to choose proper parameter values for the window-size, log-ratio threshold, and B-allele fraction threshold. To do so, plot sub-command breaks the genome into bins and plots coverage log-ratios against B-allele fractions for each bin. Each point in the plot represents one bin and its area is proportional to the number of heterozygous SNPs detected within that bin. The bin size is calculated by using the window-size provided by the user and the resolution at which the read coverage was extracted. Interpretation of the plot guides the user to choose the log-ratio threshold and B-allele fraction threshold. Figure S9 represents such a plot made from a low grade glioma sample (i.e. TCGA-CS-5395) used in this study. Based on this figure if window-size 11 kbp is used, the parameter values for log-ratio threshold and B-allele fraction threshold can be set to 0.7 and 0.2 respectively.

For other parameters, empirically derived values are set as default in Segmentum’s user interface. These values should be suitable for many of the WGS samples. However, following are some guides on selecting some of the parameters in case the results are not satisfactory. Minimum mapping quality score (i.e. --quality) by default has been set to 10; to have more reliable RD and BAF estimates, it can be increased. Minimum number of reads from the normal sample to calculate the coverage log-ratio (--min_read) has been set to 50 by default. For samples with low average coverage, this parameter can be lowered. Minimum evidence for heterozygous (i.e. --hetz=N:R) can be made more stringent or more relaxed by increasing/decreasing the number of reads having the alternate allele and its proportion to the number of reads having the reference allele. In case the segmentation result looks over-segmented, the default values for --logr_merge and --baf_merge parameters may be increased accordingly. To call copy-neutral LOH regions, default values for parameters --clogr_thresh and --baf_thresh should enable the tool to detect copy-neutral LOH events for samples with even ~50% of tumor purity. In case user knows the tumor purity beforehand, these parameters can be adjusted accordingly.


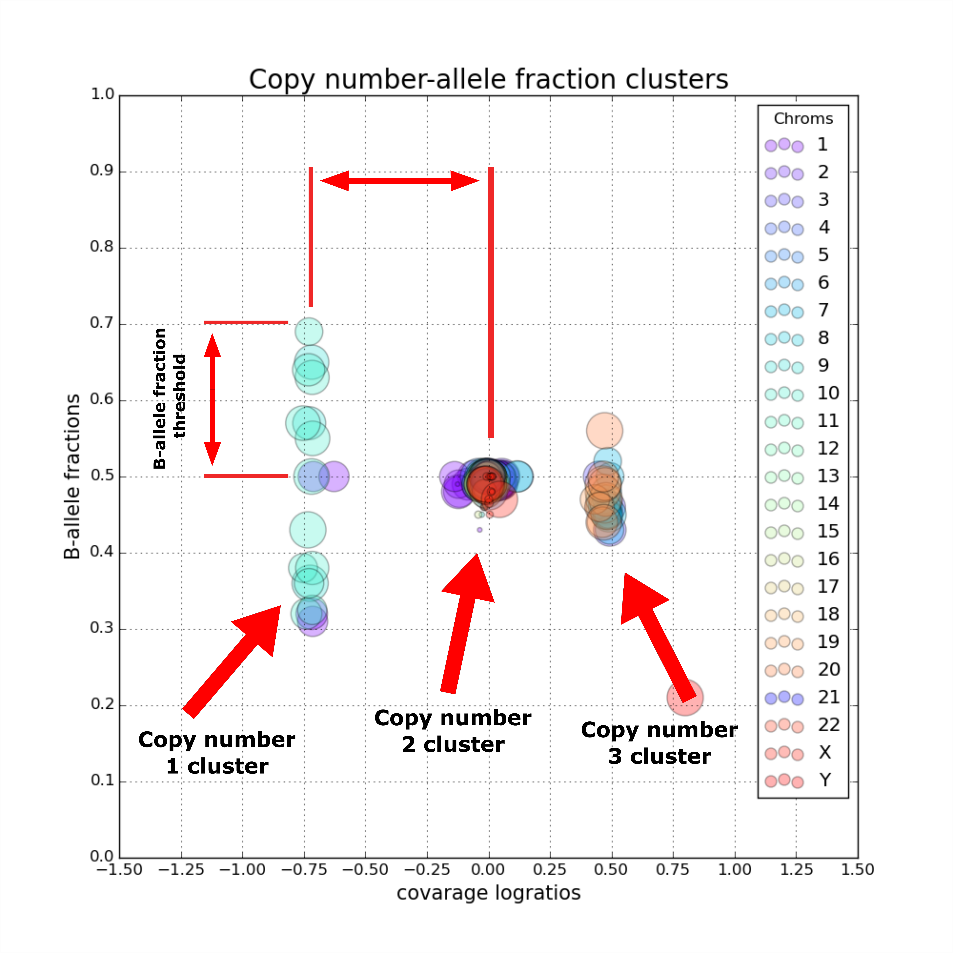


**Figure S9.** Copy number – B-allele fraction clusters. Different colors represent different chromosomes. Each circle represents one bin. The size of the circle is proportional to the number of heterozygous SNPs within that bin.

Sample barcode names

The following tables present the sample barcode names for the two TCGA datasets used in this study.

**Table S3.** TCGA LGG sample barcode names and the estimated sample purity by ABSOLUTE.

| **sample barcode names** | **Estimated sample purity by ABSOLUTE [5]** |
| --- | --- |
| TCGA-HT-7689-01A-11D-2253-08 | 0.72 |
| TCGA-DB-5278-01A-01D-1468-08 | 0.78 |
| TCGA-DU-7301-01A-11D-2086-08 | 0.88 |
| TCGA-DU-5872-01A-11D-A465-08 | 0.87 |
| TCGA-DU-5874-01A-11D-1705-08 | 0.96 |
| TCGA-CS-5395-01A-01D-1468-08.1 | 0.81 |
| TCGA-DU-6401-01A-11D-1705-08.2 | 0.37 |
| TCGA-DU-7013-01A-11D-A461-08.1 | 0.96 |
| TCGA-DU-7304-01A-12D-A461-08.4 | 0.80 |
| TCGA-FG-8182-01A-11D-2253-08.3 | 0.25 |

**Table S4.** TCGA PRAD sample barcode names and the estimated sample purity by ABSOLUTE.

| **sample barcode names** | **Estimated sample purity by ABSOLUTE [6]** |
| --- | --- |
| TCGA-G9-6332-01A-11D-1785-01 | - |
| TCGA-G9-6338-01A-12D-1959-01 | - |
| TCGA-G9-6342-01A-11D-1959-01 | 0.48 |
| TCGA-G9-6362-01A-11D-1785-01 | - |
| TCGA-G9-6364-01A-21D-1785-01 | 0.59 |
| TCGA-G9-6373-01A-11D-1785-01 | - |
| TCGA-G9-6494-01A-11D-1785-01 | 0.57 |
| TCGA-HI-7171-01A-12D-2112-01 | 0.88 |
| TCGA-HC-7211-01A-11D-2112-01 | 0.69 |
| TCGA-EJ-7784-01A-11D-2112-01 | 0.68 |

**Table S5.** TCGA LGG sample barcode names categorized based on inferred subtype.

| *IDH* mutant, 1p/19q co-del | TCGA-CS-6668-01A-11D-1893-08  TCGA-DB-5278-01A-01D-1468-08  TCGA-DH-A669-01A-12D-A31L-08  TCGA-DU-5870-01A-11D-A461-08  TCGA-DU-5874-01A-11D-1705-08  TCGA-DU-6397-01A-11D-A461-08  TCGA-DU-7009-01A-11D-2024-08  TCGA-E1-5318-01A-01D-1468-08  TCGA-E1-5319-01A-01D-1893-08  TCGA-EZ-7264-01A-11D-2024-08  TCGA-FG-5964-01A-11D-1705-08  TCGA-HT-7695-01A-11D-2253-08  TCGA-HW-7486-01A-11D-2024-08  TCGA-HW-7487-01A-11D-2024-08 |
| --- | --- |
| *IDH* mutant, no 1p/19q co-del, 17p copy-neutral loss of heterozygosity | TCGA-CS-6665-01A-11D-1893-08  TCGA-DU-5872-01A-11D-A465-08  TCGA-DU-6407-01A-13D-1705-08  TCGA-DU-7301-01A-11D-2086-08  TCGA-FG-5965-01B-11D-1893-08  TCGA-HT-7689-01A-11D-2253-08  TCGA-HT-A5R7-01A-11D-A461-08  TCGA-HT-A61B-01A-11D-A461-08  TCGA-IK-7675-01A-11D-2086-08  TCGA-TQ-A7RK-01A-11D-A33T-08  TCGA-TQ-A7RV-01A-21D-A34A-08  TCGA-TQ-A8XE-01A-11D-A36O-08 |
| *IDH* mutant, no 1p/19q co-del, no 17p copy-neutral loss of heterozygosity | TCGA-DU-6401-01A-11D-1705-08  TCGA-DU-7304-01A-12D-A461-08  TCGA-FG-8182-01A-11D-2253-08  TCGA-FG-A4MT-01A-11D-A461-08  TCGA-HT-7602-01A-21D-2086-08  TCGA-TM-A7CF-01A-11D-A32B-08 |
| *IDH* wild-type | TCGA-DU-7013-01A-11D-A461-08  TCGA-CS-5395-01A-01D-1468-08  TCGA-CS-6669-01A-11D-1893-08  TCGA-DU-6404-01A-11D-A461-08  TCGA-FG-7643-01A-11D-A461-08  TCGA-HT-8104-01A-11D-A461-08 |

Tool versions and parameter values used for the different tools

In this study, TCGA LGG and TCGA PRAD samples were analyzed with other tools using default or recommended parameter values from the tool’s manual by assuming that the developers had better ideas regarding which parameter values increased the tools’ performances.

CLImAT

DFExtract version 1.2.2 was used for extracting the read-depth and CLImAT version 1.2.2 was used to perform the analysis in this study. Default parameter values were used for running DFExtract and CLImAT (Tables S6 and S7).

**Table S6.** Parameter values for running DFExtract.

| **Options** | **Description** | **value** |
| --- | --- | --- |
| -w, --window | set the size of windows | 1000 |
| -Q, --baseQ | threshold value for base quality | 10 |
| -q, --mapQ | threshold value for mapping quality | 20 |
| -d, --minDepth | minimum read-depth for a position to be considered | 10 |

**Table S7.** Parameter values for running CLImAT.

| **Parameters** | **Description** | **Value** |
| --- | --- | --- |
| minDepth | minimum read-depth for a position to be considered | 10 |
| maxDepth | maximum read-depth for a position to be considered | 300 |
| minGC | minimum GC-content for a position to be considered | 0 |
| minMapScore | minimum mapability score for a position to be considered | 0 |
| maxMapScore | maximum mapability score for a position to be considered | 0.98 |

Patchwork

Patchwork version 2.4 was used in this study. Parameter values for the samples were manually determined from intermediate plots produced by Patchwork. Tables S8 and S9 show the parameter values for the 10 TCGA LGG and 10 TCGA PRAD samples, respectively.

**Table S8.** Parameter values for running Patchwork for 10 TCGA LGG samples.

| **Samples/Parameters** | **cn2** | **delta** | **het** | **hom** |
| --- | --- | --- | --- | --- |
| TCGA-DB-5278-10A-01D-1468-08.1 | 1.02 | 0.4 | 0.26 | 0.45 |
| TCGA-DU-5872-10A-01D-A465-08.1 | 1.02 | 0.4 | 0.22 | 0.75 |
| TCGA-DU-5874-10A-01D-1705-08.2 | 1.02 | 0.5 | 0.28 | 0.47 |
| TCGA-DU-7301-10A-01D-2086-08.2 | 1.00 | 0.41 | 0.28 | 0.59 |
| TCGA-HT-7689-10A-01D-2253-08.5 | 1.05 | 0.35 | 0.28 | 0.78 |
| TCGA-CS-5395-01A-01D-1468-08.1 | 0.98 | 0.39 | 0.28 | 0.48 |
| TCGA-DU-6401-01A-11D-1705-08.2 | 1.04 | 0.16 | 0.22 | 0.53 |
| TCGA-DU-7013-01A-11D-A461-08.1 | 0.94 | 0.42 | 0.21 | 0.55 |
| TCGA-DU-7304-01A-12D-A461-08.4 | 0.95 | 0.40 | 0.22 | 0.84 |
| TCGA-FG-8182-01A-11D-2253-08.3 | 1.00 | 0.40 | 0.21 | 0.43 |

**Table S9.** Parameter values for running Patchwork for 10 TCGA PRAD samples.

| **Samples/Parameters** | **cn2** | **delta** | **het** | **hom** |
| --- | --- | --- | --- | --- |
| TCGA-G9-6332-01A-11D-1785-01 | 1.02 | 0.08 | 0.40 | 0.50 |
| TCGA-G9-6338-01A-12D-1959-01 | 1.00 | 0.20 | 0.40 | 0.58 |
| TCGA-G9-6342-01A-11D-1959-01 | 1.00 | 0.22 | 0.40 | 0.50 |
| TCGA-G9-6362-01A-11D-1785-01 | 0.89 | 0.11 | 0.45 | 0.78 |
| TCGA-G9-6364-01A-21D-1785-01 | 1.00 | 0.10 | 0.41 | 0.47 |
| TCGA-G9-6373-01A-11D-1785-01 | 1.00 | 0.10 | 0.40 | 0.51 |
| TCGA-G9-6494-01A-11D-1785-01 | 1.20 | 0.28 | 0.39 | 0.52 |
| TCGA-HI-7171-01A-12D-2112-01 | 1.05 | 0.15 | 0.45 | 0.60 |
| TCGA-HC-7211-01A-11D-2112-01 | 1.01 | 0.22 | 0.37 | 0.46 |
| TCGA-EJ-7784-01A-11D-2112-01 | 1.00 | 0.50 | 0.40 | 0.58 |

Control-FREEC

Control-FREEC version 7.2 was used in this study. Table S10 shows the common parameter values used to run Control-FREEC for both the TCGA LGG and PRAD samples.

**Table S10.** Parameter values for running Control-FREEC.

| **Parameter** | **Parameter value** |
| --- | --- |
| **[general]** | |
| coefficientOfVariation | 0.05 |
| contaminationAdjustment | TRUE |
| intercept | 1 |
| breakPointType | 4 |
| ploidy | 2 |
| sex | XY |
| numberOfProcesses | 5 |
| **[sample]** | |
| inputFormat | pileup |
| mateOrientation | FR |
| **[control]** | |
| inputFormat | pileup |
| mateOrientation | FR |
| **[BAF]** | |
| SNPfile | hg19_snp138_1based.txt |

References

1. Boeva V, Popova T, Bleakley K, Chiche P, Cappo J, Schleiermacher G, Janoueix-Lerosey I, Delattre O, Barillot E: Control-FREEC: a tool for assessing copy number and allelic content using next-generation sequencing data. *Bioinformatics* 2012, 28(3)**;**423-425.

2. Liu B, Morrison CD, Johnson CS, Trump DL, Qin M, Conroy JC, Wang J, Liu S: Computational methods for detecting copy number variations in cancer genome using next generation sequencing: principles and challenges. *Oncotarget* 2013, 4(11)**;**1868-1881.

3. Mayrhofer M, DiLorenzo S, Isaksson A: Patchwork: allele-specific copy number analysis of whole-genome sequenced tumor tissue. *Genome Biol* 2013, 14(3)**;**R24-2013-14-3-r24.

4. Yu Z, Liu Y, Shen Y, Wang M, Li A: CLImAT: accurate detection of copy number alteration and loss of heterozygosity in impure and aneuploid tumor samples using whole-genome sequencing data. *Bioinformatics* 2014, **;**1-8.

5. Cancer Genome Atlas Research Network: Comprehensive, Integrative Genomic Analysis of Diffuse Lower-Grade Gliomas", *The New England journal of medicine,* 2015,  372(26); 2481-2498.

6. Cancer Genome Atlas Research Network: "The Molecular Taxonomy of Primary Prostate Cancer", *Cell,*2015, 163(4); 1011-1025.
